# Bad Prognosis in Critical Ill Patients with COVID-19 during Short-Term ICU Stay regarding Vitamin D Levels

**DOI:** 10.3390/nu13061988

**Published:** 2021-06-09

**Authors:** Lourdes Herrera-Quintana, Yenifer Gamarra-Morales, Héctor Vázquez-Lorente, Jorge Molina-López, José Castaño-Pérez, Juan Francisco Machado-Casas, Ramón Coca-Zúñiga, José Miguel Pérez-Villares, Elena Planells

**Affiliations:** 1 Department of Physiology, Institute of Nutrition and Food Technology “Jose Mataix”, School of Pharmacy, University of Granada, 18071 Granada, Spain; lourdesherrera@ugr.es (L.H.-Q.); jennifer_gamo@hotmail.com (Y.G.-M.); hectorvazquez@ugr.es (H.V.-L.); 2 Faculty of Education, Psychology and Sports Sciences, University of Huelva, 21007 Huelva, Spain; 3 Intensive Care Unit, Virgen de las Nieves Hospital, Fuerzas Armadas Avenue, 18014 Granada, Spain; jcastaoperez@yahoo.com (J.C.-P.); jmachc2@hotmail.com (J.F.M.-C.); ramon.coca.sspa@juntadeandalucia.es (R.C.-Z.); josem.perez.villares.sspa@juntadeandalucia.es (J.M.P.-V.)

**Keywords:** coronavirus disease 2019, SARS-CoV-2, Vitamin D, critical care, intensive care patient

## Abstract

Background and aims: Vitamin D inadequacy may be involved in the mechanisms of SARS-CoV-2 infection and in potential risk factors for disease propagation or control of coronavirus disease 2019 (COVID-19). This study assessed a short-term evolution of vitamin D status and its influence upon different clinical parameters in critically ill patients with COVID-19. Methods: A prospective analytical study in which 37 critically ill volunteers between 41 and 71 years of age with COVID-19 were evaluated at baseline and three days of intensive care unit (ICU) stay. 25-OH-D_3_ and 25-OH-D_2_ were analyzed by liquid chromatography–tandem mass spectrometry and total 25-OH-D levels were calculated as the sum of both. Results: All patients presented low 25-OH-D levels at baseline, decreasing total 25-OH-D (*p* = 0.011) mainly through 25-OH-D_2_ (*p* = 0.006) levels during ICU stay. 25-OH-D_2_ levels decreased a mean of 41.6% ± 89.6% versus 7.0% ± 23.4% for the 25-OH-D_3_ form during the ICU stay. Patients who did not need invasive mechanical ventilation presented higher levels of 25-OH-D_2_ at baseline and follow-up. Lower 25-OH-D and 25-OH-D_3_ levels were associated with higher D-dimer at baseline (*p* = 0.003; *p* = 0.001) and at follow up (*p* = 0.029), higher procalcitonin levels (*p* = 0.002; *p* = 0.018) at follow up, and lower percentage lymphocyte counts (*p* = 0.044; *p* = 0.040) during ICU stay. Conclusions: Deficient vitamin D status in critical patients was established at the admission and further worsened after three days of stay. Lower vitamin D levels were related to key altered clinical and biochemical parameters on patients with SARS-CoV-2 infection. Given the different response of the 25-OH-D_3_ and 25-OH-D_2_ forms, it would be useful to monitor them on the evolution of the critically ill patient.

## 1. Introduction

The global public health crisis caused by the SARS-CoV-2 virus has created the need for urgent actions in order to reduce the risk of infection, progression, and the severity of coronavirus disease 2019 (COVID-19) [1], which triggers an acute inflammatory process and uncontrolled oxidative stress [2]. This in turn results in severe acute respiratory distress syndrome (ARDS) characterized by a cytokine storm, mainly in critical cases [3], which may lead to multiple organ damage [4] and further complicate the patient’s critical condition previously described during their ICU stay [5,6].

There is currently great concern regarding the clinical management and intensive care of patients with critical stages of the disease and who are at a higher risk of death [7]. The implementation of prompt and appropriate nutritional assessment in COVID-19 must be considered, [8,9] because possible modulation of the status of key micronutrients appears to be a relevant factor influencing the development of this disease [10]. No information about nutritional monitoring in critical patients with COVID-19 is available to date [11], and this lack of data precludes the definition of firm micronutrient recommendations in this particular risk population [12].

Certain micronutrients are essential for adequate immunocompetence and antioxidant defense, which are related to inflammatory response, such as vitamin D [13]. 25-Hydroxyvitamin D (25-OH-D) is the metabolite used to assess vitamin D status, due to its long half-life in plasma or serum (one month) [14], and is characterized by synergic action of its two main forms: 25-hydroxyvitamin D_2_ (25-OH-D_2_), which is obtained from plant sources, and 25-hydroxyvitamin D_3_ (25-OH-D_3_), which comes from animal products and endogenous synthesis in skin through exposure to sunlight [15], both of them can be supplemented with commercial products [16]. Recently, vitamin D has generated particular interest because of its role in reducing the risk of pneumonia and viral upper respiratory tract infections at a physical barrier and cellular natural and adaptive immunity level [17,18]. The underlying mechanisms can be grouped into two main actions: anti-inflammatory and anti-infective [19]. Vitamin D is associated with a decrease in proinflammatory cytokines, reducing the cytokine storm induced by the innate immune system, which is exacerbated in COVID-19 [17,20]. Moreover, it must be noted that serum 25-OH-D is considered as a negative acute phase reactant [21] and low vitamin D status in critical ill patients may be related to a decrease of binding protein concentration [22]. On the other hand, vitamin D is able to reduce viral infection and replication rates by inducing transcription of proteins with antimicrobial functions, enhancing autophagic encapsulation of viral particles, favoring lung epithelial cell barrier integrity, and ultimately regulating both innate and adaptive immunity [17,18,19,20,23,24].

Thus, vitamin D inadequacy has emerged as a factor that may be involved in the mechanisms of virus infection and in potential risk factors for disease propagation or control [24,25]. In fact, low vitamin D status in patients with COVID-19 has been reported [26,27,28], being associated with a poorer prognosis, infection risk, or unregulated inflammation. Thus, due to the lack of evidence on the importance of monitoring vitamin D status in critical patients with COVID-19, the present study was designed to assess the short-term evolution of the status of vitamin D and its influence upon different clinical parameters in critically ill patients with COVID-19 in the province of Granada, Spain.

## 2. Materials and Methods

### 2.1. Study Design and Subjects

A prospective analytical study was carried out of patients monitored from the first day of admission to the intensive care unit (ICU) (baseline) until day three of stay (follow-up). Of a total of 43 initially recruited patients, 37 participants from the province of Granada (Spain), aged 41–74 years, were included in the period from 1 March to 1 June 2020, after been informed about the study protocol. Six patients died during the study and were excluded. All eligible participants enrolled in the study were critical patients aged 18 years or older and hospitalized for more than 48 h, who agreed to participate in the study or for whom approval of participation was obtained from the family. All patients had a diagnosis of critical active SARS-CoV-2 infection according to the Chinese Clinical Guideline for the classification of COVID-19 [29] (analyzed by real-time reverse transcriptase–PCR (RT-PCR)) testing of nasal and pharyngeal swab samples) and had an ICU stay of at least three days and did not receive vitamin D support. The present study was conducted in accordance with the principles of the Declaration of Helsinki, following the International Conference on Harmonization/Good Clinical Practice standards, and was approved by the Ethics Committee of the University of Granada (Ref. 149/CEIH/2016).

### 2.2. Data Collection

Data including age, sex, body mass index (BMI), smoking habits, comorbidities, respiratory and clinical parameters, ICU length of stay, length of hospitalization, and 28-day mortality were retrieved from the hospital electronic database system and recorded for each study participant at ICU admission (baseline) and after three days (follow-up). The Acute Physiology and Chronic Health Evaluation II (APACHE-II) score and Sequential Organ Failure Assessment (SOFA) score were obtained by intensivists at baseline and follow-up.

Patient clinical outcomes were recorded both at admission and during the ICU stay: heart rate (beats per minute); respiratory rate (breaths per minute); mean blood pressure (mmHg); positive end-expiratory pressure (PEEP); fraction of inspired oxygen (FiO_2_); partial oxygen arterial pressure/fraction of inspired oxygen (PaO_2_/FiO_2_); ARDS; invasive mechanical ventilation (IMV).

### 2.3. Blood Sampling and Biochemical Parameters

Two measurements were performed (baseline and follow-up). Blood sampling was carried out in the morning under fasting conditions, followed by centrifugation (4 °C for 15 min at 3500 rpm) to separate the plasma. The samples were frozen at −80 °C until analysis of the different parameters. All samples were measured in one run, in the same assay batch, and blinded quality control samples were included in the same assay batches to determine laboratory error in the measurements.

The recorded biochemical parameters were total proteins, albumin, prealbumin, ferritin, transferrin, glucose, total cholesterol, glutamic oxaloacetic transaminase or aspartate transaminase (GOT or AST), glutamic pyruvic transaminase or alanine transaminase (GPT or ALT), lactate dehydrogenase (LDH), C-reactive protein (CRP), procalcitonin (PCT), hemoglobin, leukocytes, neutrophils, lymphocytes, platelets, D-dimer, fibrinogen, calcium (Ca), phosphorous (P), and magnesium (Mg), using routine hospital analytical assays (ECLIA, Elecsys 2010 and Modular Analytics E170, Roche Diagnostics, Mannheim, Germany).

### 2.4. Analytical Determination of Vitamin D

Vitamin D was measured in plasma samples by liquid chromatography–tandem mass spectrometry (LC-MS/MS). Plasma sample treatment involved protein precipitation adding 500 μL of acetonitrile in an Eppendorf flask with 200 μL of plasma and 20 μL of 25-hydroxyvitamin D_3_ and 25-hydroxyvitamin D_2_ deuterated solutions as Internal Standard (IS) (Sigma Aldrich, St. Louis, MO, USA) (0.5 μg/mL). The samples were slightly shaken for 1 min on a plate shaker and centrifuged at 10,000 rpm for 15 min at 4 °C. The supernatant was collected in another Eppendorf flask and dried with N_2_. The dry residue was vortexed for 30 s after the addition of 200 μL of ethyl acetate and 100 μL of deionized water and centrifuged at 3000 rpm for 5 min. The supernatant was again collected in another Eppendorf flask, and the previous steps were repeated with the remaining liquid phase, subsequently pooling the second supernatant with the first. The total supernatant was dried with N_2_.

For samples derivatization, we prepared a solution of 4-phenyl-3H-1,2,4-triazole-3,5(4H)-dione (PTAD) (Sigma Aldrich, St. Louis, MO, USA) in acetonitrile (0.5 mg/mL), using 50 μL of this solution in standards and in each sample, with vortexing. All samples were placed on the plate shaker for 1 h at room temperature and covered with aluminum foil. Lastly, the samples were transferred to vials, diluted with 50 μL of deionized water and stored in freezer at −20 °C covered with aluminum foil until injection into the chromatograph. For the calibration line, increasing concentrations of 1, 2, 5, 10, 25, 50, and 100 ppb of the standards 25-OH-D_3_ and 25-OH-D_2_ (Sigma Aldrich, St. Louis, MO, USA) with 20 μL of IS were used and dried with N_2_ and derivatized at the same time as the samples. For sample measurements, use was made of a Waters Acquity UHPLC I-Class System chromatograph (Waters, London, UK), with the Acquity UHPLC BEH C18 column 2.1, 50 mm, 1.7 m at room temperature. The mobile phase of channel A was water with 50 mM of ammonium formate, while that of channel B was methanol. The injection volume of the sample was 10 μL and the flow rate was 0.4 mL/min. The detector was a Waters XEVO-TQ-XS Triple Quadrupole Low Resolution Spectrometer. Total 25-OH-D was calculated as the sum of the 25-OH-D_3_ and 25-OH-D_2_ forms. According to the Endocrine Society Practice Guidelines on Vitamin D, the threshold for biochemical 25-OH-D sufficiency values was considered to be >30 ng/mL, with deficiency being defined as 20–29 ng/mL and insufficiency as <20 ng/mL [28].

### 2.5. Statistical Analysis

Qualitative variables were presented as frequencies and percentages. Quantitative variables with normal distribution were expressed as the arithmetic mean and standard deviation (SD), and variables with non-normal distribution were expressed as the median and the interquartile range. Normal data distribution for continuous variables was tested using the Shapiro–Wilk test. The Wilcoxon signed-rank test for nonparametric samples was used for the comparative analyses at baseline and follow-up. The unpaired Student *t*-test for parametric samples was used for the comparative analysis based on clinical outcomes. The effect size (ES) was estimated and interpreted as follows: small = 0.01, moderate = 0.06, and large = 0.14 [30]. Correlation analyses and partial correlation coefficients were performed using the Spearman test. Statistical significance was considered for *p* < 0.05. The SPSS version 22.0 statistical package (IBM SPSS, Armonk, NY, USA) was used throughout.

## 3. Results

The demographic and clinical characteristics of the 37 patients enrolled in the study are shown in Table 1. The median age of the study population was 60 years, and the gender distribution of the sample was 26 males and 11 females. With regard to the anthropometric parameters, over a third of the patients were overweight, and more than half of them were obese. Most of the patients had one or more underlying diseases. With regard to the severity parameters, the mean APACHE-II and SOFA scores were 12.3 and 6.54, respectively, upon admission. A total of 26 of 37 patients (70.2%) had at least 1 infection, and 5 of them had 3 or more infections during their admission in ICU. The most frequent infection was bacteremia in 25 out of 48 total infections (52%), followed by respiratory infections 15 out of 48 total infections (31%), and finally urinary tract infections associated with urethral catheterization in 7 out of 48 total infections (14.5%). The most frequent germs causing these infections were Gram-positive (39%), followed by Gram-negative bacteria (33%) and fungi (20%). The mean length of hospitalization was 39.5 days. More than two-thirds of the patients presented ARDS on admission, requiring invasive mechanical ventilation with a mean duration of over 20 days. The mortality rate after 28 days of ICU stay was over two-thirds of the total study population.

The clinical and biochemical parameters of the study population at baseline and follow-up are shown in Table 2. Respiratory parameters were altered with significant changes in FiO_2_ and PEEP after three days. Total proteins (*p* = 0.012), albumin (*p* = 0.035), prealbumin (*p* = 0.017), LDH (*p* = 0.002), CRP (*p* = 0.001), hemoglobin (*p* = 0.001), and fibrinogen (*p* = 0.001) were outside the reference values and decreased significantly after three days of ICU stay. Parameters such as ferritin, transaminases, or D-dimer were also outside the reference values although no changes were observed in their evolution during the ICU stay. The results showed the 25-OH-D, 25-OH-D_3_, and 25-OH-D_2_ levels to be lower at follow-up versus baseline—with statistical significance being reached for 25-OH-D (*p* = 0.011) and 25-OH-D_2_ (*p* = 0.006).

**Figure 1:**
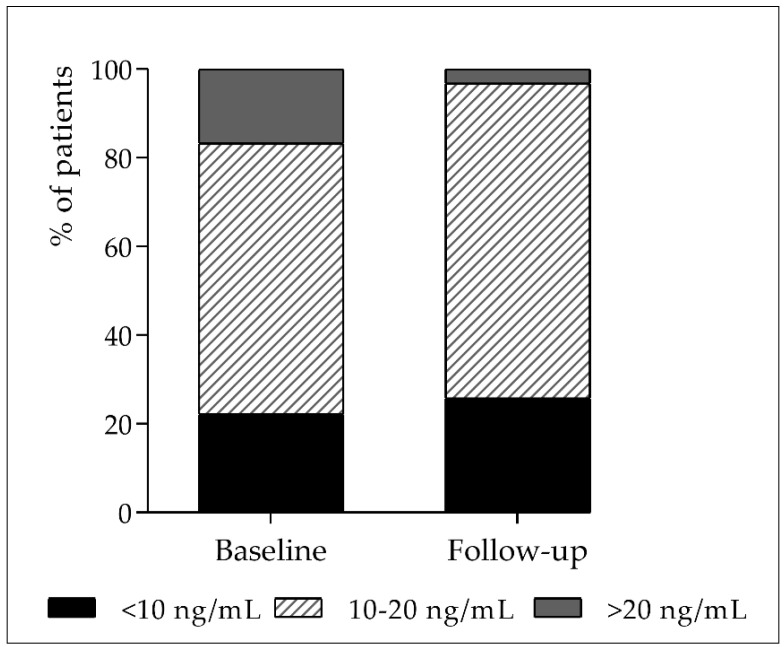
Vitamin D status in the study population at ICU admission (baseline) and after three days (follow-up).

Figure 1 shows the distribution of patient vitamin D status upon ICU admission and after three days of stay. In no case were the 25-OH-D levels > 25 ng/mL. Only 16.7% (6/37) of the patients had 25-OH-D > 20 ng/mL at baseline, versus 3.2% (1/37) at follow-up. Furthermore, 22.2% (8/37) presented 25-OH-D < 10 ng/mL—this percentage reaching 25.8% (10/37) after three days of stay.

**Figure 2:**
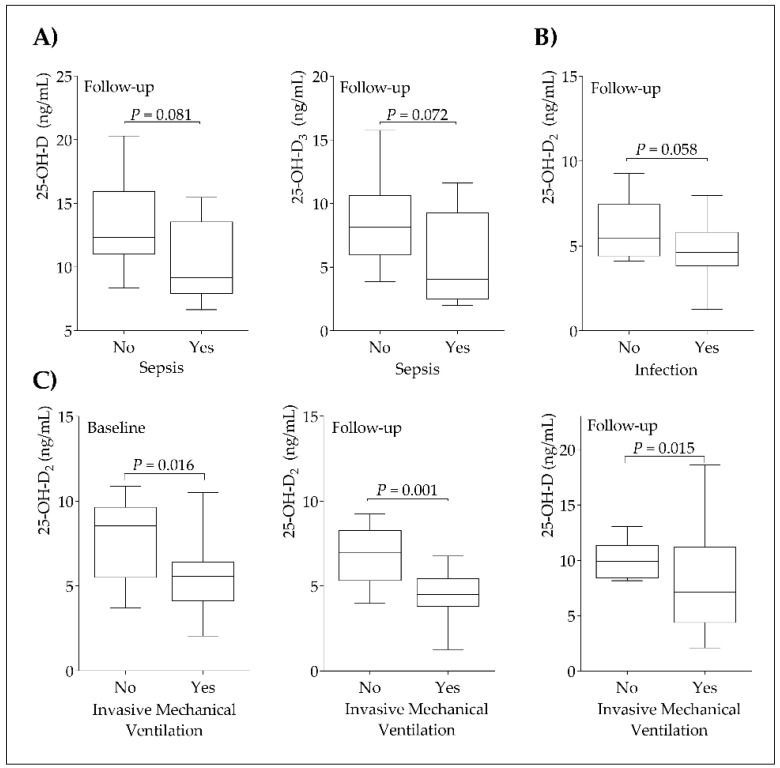
Comparative analysis of septic and non-septic patients with 25-OH-D and 25-OH-D_3_ levels at follow-up (**A**); infection with 25-OH-D_2_ levels at follow-up (**B**); invasive mechanical ventilation with 25-OH-D_2_ levels at baseline and 25-OH-D_2_ and 25-OH-D levels at follow-up (**C**). Statistical significance = *p* < 0.05.

Figure 2 corresponds to the comparative analysis of clinical parameters in relation to 25-OH-D, 25-OH-D_3_, and 25-OH-D_2_ levels upon ICU admission and after three days of stay. Based on the sepsis (Figure 2A) and infectious processes (Figure 2B), there was a trend toward statistical significance in the follow-up observing lower levels of 25-OH-D and 25-OH-D_3_ for septic patients and lower levels of 25-OH-D_2_ for infected patients. Patients who did not need invasive mechanical ventilation presented higher levels of 25-OH-D_2_ at baseline and 25-OH-D and 25-OH-D_2_ at follow-up (Figure 2C).

Table 3 shows the Spearman bivariate correlations between the 25-OH-D, 25-OH-D_3_, and 25-OH-D_2_ levels at baseline and follow-up and the clinical and biochemical parameters analyzed in our study. At baseline, the 25-OH-D levels were correlated to albumin (*p* = 0.021), hemoglobin (*p* = 0.028), D-dimer (*p* = 0.003), and fibrinogen (*p* = 0.020)—with albumin also being correlated to 25-OH-D_2_ (*p* = 0.037); and D-dimer (*p* = 0.001) and fibrinogen to 25-OH-D_3_ (*p* = 0.029). Respiratory rate was negatively correlated to 25-OH-D_2_ (*p* = 0.025). At follow-up, 25-OH-D and 25-OH-D_3_ were significantly correlated to PCT (*p* = 0.002; *p* = 0.018) and lymphocytes (*p* = 0.044; *p* = 0.040). In the case of D-dimer and Ca, an inverse correlation to 25-OH-D_3_ was observed (*p* = 0.029 and *p* = 0.006, respectively). Finally, the 25-OH-D_2_ levels showed a significant correlation to both the fibrinogen (*p* = 0.003) and Ca levels (*p* = 0.030). We did not find correlation between mortality rate after 28 days of ICU stay and 25-OH-D_3_, 25-OH-D_2_, and 25-OH-D levels.

## 4. Discussion

The main finding of the present study was the low 25-OH-D levels in the patients upon admission (baseline), followed by a significant decrease after three days of ICU stay. The entire population was below the sufficiency reference values for 25-OH-D, and most of them presented insufficient 25-OH-D status. We also analyzed the 25-OH-D_3_ and 25-OH-D_2_ levels, both of which were seen to decrease after three days (though statistical significance was only reached in the case of 25-OH-D_2_), thus influencing upon 25-OH-D decreased levels and presenting a worsening during their stay at 3 days. Moreover, vitamin D was associated with clinical parameters such as the need for mechanical ventilation or respiratory frequency and with biochemical parameters also associated with the severity of the critically ill patient such as albumin, hemoglobin, D-dimer, fibrinogen, PCT, and lymphocytes.

Previous evidence points to poorer COVID-19 outcomes associated with factors such as the male gender, older age, BMI > 35 kg/m^2^, and the presence of certain comorbidities [31]. The demographic and clinical characteristics of our patients (Table 1) are consistent with this evidence. In effect, the population was fundamentally elderly, two-thirds were males, and there was a high prevalence of comorbidities and obesity. It should be noted that metabolically ill patients with obesity may have a high risk of suffering inflammatory processes [32], which could contribute to a greater probability of poorer outcomes.

Many of the clinical and biochemical parameters in our patients were altered (Table 2). In the case of PEEP and FiO_2_, levels were above reference values and, they even decreased significantly in three days and remained altered in the most cases. It should be noted that ferritin, CRP, D-dimer, and fibrinogen were well above the reference values. Parameters related to inflammation (such as CRP) or coagulation (such as D-dimer) have been correlated to a poor prognosis and have been described as possible predictive biomarkers of COVID-19 [33].

Our results with reference to vitamin D status showed all patients to have insufficient levels (<30 ng/mL) both upon admission and during the study period—with the vitamin D status being seen to worsen in only three days. The great majority of patients presented vitamin D deficiency (<20 ng/mL), while extreme deficient values of <10 ng/mL were recorded in a quarter of the study sample. It is known that, compared to the general population, the prevalence of hypovitaminosis D is greater in the critically ill and may constitute a risk factor for adverse outcomes [34]. Moreover, these levels could be influenced by seasonality. In an observational study carried out in critical ill patients from Austria, significant differences were noted in the prevalence of vitamin D deficiency and in the mean 25-OH-D values between the winter and summer months [35]. Our study covered the period from March to June; we, therefore, could not demonstrate the influence of seasonality in our patients.

On the other hand, there is concern about the high prevalence of hypovitaminosis D in the general population—being regarded as a global health issue with important consequences [36]. Moreover, SARS-CoV-2 positivity has been strongly and inversely correlated to circulating 25-OH-D levels—a relationship that persists across latitudes, races/ethnicities, both genders, and age ranges [37], thereby evidencing that the COVID-19 fatality rates parallel the vitamin D deficiency rates [38]. These negative correlations between vitamin D deficiency and the number of COVID-19 cases and mortality have also been reported in another 20 European countries [39]. It could be expected that countries such as Spain have a better vitamin D status and therefore less severe consequences than other countries in northern Europe. However, our results reinforced the evidence of a possible widespread vitamin D deficiency in the Spanish population. Indeed, vitamin D deficiency in Italy and Spain (the countries presenting the highest age-specific case fatality ratio) [40] is more severe than elsewhere in Europe [41], particularly in the aging population [39].

On the other hand, Maghbooli et al. found 25-OH-D levels >30 ng/mL to reduce the risk of adverse clinical outcomes in patients with COVID-19 [42]. None of our critical patients with COVID-19 presented 25-OH-D levels >25 ng/mL, which is consistent with the findings of Maghbooli. Low 25-OH-D levels have been reported by many authors in hospitalized patients with COVID-19 [43], with such deficiency being associated with a greater mortality risk [26]. In fact, Vassiliou et al. found that low 25-OH-D levels in patients with COVID-19 at ICU admission could predispose to an increased 28-day mortality risk [44]. In the present study, although we did not observe an association between 25-OH-D levels with 28-day mortality risk, we found an association with clinical outcomes, reporting that higher 25-OH-D and 25-OH-D_2_ levels were associated with those patients who did not require invasive mechanical ventilation. Moreover, lower 25-OH-D values, as a result of decreased 25-OH-D_3_ and 25-OH-D_2_ levels were observed with the presence of infection (bacterial/fungal) and sepsis (Figure 2). It should be noted that 25-OH-D_2_ levels were in agreement with previous studies in the Spanish population [45], being also similar to those found in other studies performed in Belgian and Chinese populations [46,47]. Likewise, observational studies have also shown that higher 25-OH-D levels would be associated with better clinical outcomes in respiratory diseases [48]. Nevertheless, reference values are needed to have more contrastable evidence on 25-OH-D_2_ levels.

In relation to infection, a high percentage of our patients (70.3%) showed bacteriological or fungal infection, which would support the idea that the daily risk rate of infection in COVID-critically-ill patients is increased during ICU stay [49]. Recent studies [50], consistent with the high prevalence of hypovitaminosis D observed in our study, suggest a possible role of low vitamin D in the increased risk of SARS-CoV-2 infection and subsequent hospitalization. Likewise, 25-OH-D levels were inversely associated with coagulation and sepsis, in addition to major comorbidities. Both, 25-OH-D_3_ and 25-OH-D_2_ forms tended to respond differently in patients with bacteriological or fungal infection and in patients presenting sepsis. On the other hand, it was observed that 25-OH-D_2_ levels decreased a mean of 41.6% ± 89.6% versus 7.0% ± 23.4% for the 25-OH-D_3_ form during the ICU stay, which would suggest that the lack of vitamin D support during ICU stay could have allowed this more pronounced decrease in case of the 25-OH-D_2_ form, since it would depend on the intake through diet (before the admission) or its supplementation (during ICU stay). A greater decrease in 25-OH-D_2_ relative to 25-OH-D_3_ could be also related to the lower affinity of 25-OH-D_2_ for vitamin D–binding protein, leading to a shorter half-life and a higher rate of clearance from the circulation [51], and in some cases, it even caused a decline, thereby, precipitating in vitamin D deficiency [52]. This, together with the fact that there is literature that already reports a possible different role of 25-OH-D_3_ and 25-OH-D_2_ forms [51,53], although not in critical patients, could evidence the different correlations obtained with bacterial/fungal infection or sepsis and invasive mechanical ventilation previously described. Therefore, it would suggest that a lower vitamin D status at admission and worsening during three days of ICU stay may be a modifiable risk factor and an early predictive marker of adverse outcomes in hospitalized patients with COVID-19.

On associating the concentrations of both 25-OH-D and 25-OH-D_3_ with other biochemical severity parameters, significantly lower vitamin D levels were correlated to higher D-dimer and PCT levels and a lower percentage of lymphocytes (Table 3). A recent meta-analysis has demonstrated that patients with severe COVID-19 tend to present increased leukocyte and neutrophil counts, neutrophil–lymphocyte ratio, PCT and CRP levels, and a decreased number of total lymphocytes, among parameters, compared to nonsevere individuals [54]. Furthermore, blood hypercoagulability is common among hospitalized patients with COVID-19. Elevated D-dimer levels are consistently reported in this scenario, and a gradual increase of this parameter in the course of the disease is particularly associated with patient worsening. Similarly, lower fibrinogen levels were found in nonsurvivor patients with COVID-19 [55]. In this line, the relationship between fibrinogen and 25-OH-D levels is often reported in the literature in noncritical patients [56,57]. In our study, 25-OH-D and 25-OH-D_3_ levels were inversely correlated with D-dimer levels at baseline. Furthermore, 25-OH-D_3_ levels have been correlated with fibrinogen levels at baseline. Our results may reflect a better vitamin D status (mainly due to vitamin D_3_) in patients with a more appropriate hematological profile. We observed a positive correlation at baseline between albumin (which was below the reference values) and the levels of both 25-OH-D and 25-OH-D_2_. Recently, low albumin levels have been regarded as more of a disease severity marker than as a marker of malnutrition, when such low levels are detected upon admission to hospital [58].

The present study has limitations and strengths. As limitations, the present study enrolled fewer patients than desired due to the difficulty in obtaining the sample and the patient’s own clinical situation and severity both at admission and during the ICU stay. Therefore, the data should be treated with caution in order to generalize the findings of the study. Thus, on a comparative level, the effect size was shown for a better understanding. We had no reliable data on exposure to sunlight, dietary factors, or vitamin D supplementation—all of which affect vitamin D status. The overall negative results may be related to the heterogeneity of the subjects and their underlying disease conditions or severity, which may all influence the plasma 25-OH-D levels. Our findings cannot be generalized to other populations or ethnic groups, especially considering the wide range of COVID-19 prevalence. Replicating this study in a larger, prospective, and heterogeneous population and taking into account a control group, would allow for other stratified analyses based on demographic and biochemical characteristics, taking seasonality into account, and could further corroborate our findings. Additional research is therefore needed to validate our findings. As strengths, the present study used LC-MS/MS, which is the gold standard for assessing the levels of 25-OH-D [59], affording greater sensitivity, flexibility, and specificity than the enzymoimmunoassay techniques commonly used in clinical practice, which tend to overestimate the 25-OH-D values in cases of deficiency [60,61].

Recent studies have reported encouraging results after vitamin D intervention [62,63]. However, further evidence is needed to confirm that improving vitamin D status is of benefit in reducing disease severity and mortality and the probability of developing a critical clinical condition. It is essential to ensure close patient monitoring before establishing intervention guidelines [64]. This study is one of the few that have been conducted in this context, assessing the short-term evolution of the 25-OH-D levels (through 25-OH-D_2_ and 25-OH-D_3_ levels) and its impact in critical patients with COVID-19.

## 5. Conclusions

Our data reflect a high prevalence of hypovitaminosis D in all the critical patients at ICU admission, which increased after only three days of ICU stay. On the other hand, the associations observed between 25-OH-D levels, through 25-OH-D_3_ and 25-OH-D_2_ values and key clinical outcomes and biochemical altered parameters, suggests that it might be helpful to assess vitamin D status in patients with SARS-CoV-2 infection. Given the different response of the 25-OH-D_3_ and 25-OH-D_2_ forms, it would be useful to analyze them to elucidate the role of each form on the evolution of the critically ill patient. Further investigations are needed to define underlying mechanisms in vitamin D deficiency and useful strategies based on vitamin D interventions aimed at preserving vitamin D status and enhancing the clinical and biochemical profile of critical patients with COVID-19.

## Figures and Tables

**Table 1:** Demographic and clinical characteristics of the patients.

Baseline Characteristics (*N* = 37)	Mean ± SD	Min–Max	95% CI
Age (years)	60.0 ± 10.2	41.0–74.0	56.6–63.4
Sex (M/F, %)	26/11 (70.3/29.7)	-	-
BMI (kg/m^2^)	30.77 ± 4.17	22.8–42.2	29.4–32.2
BMI < 25 kg/m^2^ (*n*/*N*, %)	3/37 (8.10)	-	-
BMI 25–30 kg/m^2^ (*n*/*N*, %)	14/37 (37.8)	-	-
BMI > 30 kg/m^2^ (*n*/*N*, %)	20/37 (54.1)	-	-
Smoking habit (*n*/*N*, %)			
Smokers	3/37 (8.10)	-	-
Ex-smokers	12/37 (32.4)	-	-
Never smokers	22/37 (59.5)	-	-
Patients with comorbidity (*n*/*N*, %)	26/37 (70.3)	-	-
Diabetes	13/37 (35.1)	-	-
Hypertension	20/37 (54.1)	-	-
Dyslipidemia	11/37 (29.7)	-	-
Chronic kidney disease	2/37 (5.40)	-	-
COPD	10/37 (27.0)	-	-
Cardiovascular disease	6/37 (16.2)	-	-
APACHE-II score	12.3 ± 3.77	6.00–21.0	11.1–13.6
SOFA score	6.54 ± 2.60	2.00–13.0	5.67–7.41
Bacterial and fungal infection (*n*/*N*, %)	26/37 (70.3)	-	-
Sepsis (*n*/*N*, %)	7/36 (19.4)	-	-
PaO_2_/FiO_2_	212.9 ± 103.8	15.0–550.0	169.2–248.6
ARDS (PaO_2_/FiO_2_ < 300) (*n*/*N*, %)	26/37 (70.0)	-	-
Mild (300 < PaO_2_/FiO_2_ ≤ 200) (*n*/*N*, %)	12/37 (32.4)	-	-
Moderate (200 < PaO_2_/FiO_2_ ≤ 100) (*n*/*N*, %)	10/37 (27.0)	-	-
Severe (PaO_2_/FiO_2_ < 100) (*n*/*N*, %)	4/37 (10.8)	-	-
IMV (*n*/*N*, %)	30/37 (81.1)	-	-
Duration of IMV (days)	21.7 ± 14.6	1.00–73.0	16.0–27.4
ICU length of stay (days)	25.4 ± 22.6	6.00–104.0	17.8–32.9
Length of hospitalization (days)	39.5 ± 27.0	9.00–131.0	30.5–48.5
Patient 28-day mortality (*n*/*N*, %)	26/37 (70.3)	-	-

*N* = 37. Data expressed as mean ± standard deviation. Abbreviations: SD = standard deviation; Min–Max = minimum–maximum; CI = confidence interval; M/F = male/female; BMI = body mass index; COPD = chronic obstructive pulmonary disease; APACHE-II = Acute Physiology and Chronic Health Evaluation II; SOFA = Sequential Organ Failure Assessment; PaO_2_/FiO_2_ = partial oxygen arterial pressure/fraction of inspired oxygen; ARDS = acute respiratory distress syndrome; IMV = invasive mechanical ventilation; ICU = intensive care unit.

**Table 2:** Clinical and biochemical parameters of the critical patients with COVID-19 at baseline and follow-up.

	Reference	Baseline Median (IQR) *N* = 37	Follow-Up Median (IQR) *N* = 37	Z	*p*-Value Initial–Final	ES
**Clinical**						
Heart rate (bpm)	60–100	80.0 (28.7)	64.0 (38.0)	−1.69	0.091	0.411
Respiratory rate (brpm)	15–20	30.0 (3.50)	22.0 (4.50)	−1.63	0.102	0.582
Mean blood pressure (mmHg)	70–105	93.5 (18.0)	91.5 (25.7)	−0.31	0.753	0.095
PEEP (cm H_2_O)	2–5	14.0 (3.50)	12.0 (2.00)	−2.76	0.006	0.779
FiO_2_	>68%	0.70 (0.25)	0.60 (0.15)	−3.81	0.001	0.825
PaO_2_/FiO_2_	200–300	200.0 (101.5)	222.0 (119.0)	−0.05	0.964	0.010
**Biochemical**						
Total Proteins (g/dL)	6.60–8.30	6.40 (0.90)	6.10 (1.13)	−2.51	0.012	0.513
Albumin (g/dL)	3.50–5.20	3.20 (0.65)	3.00 (0.60)	−2.11	0.035	0.444
Prealbumin (mg/dL)	16.0–42.0	9.00 (16.2)	25.0 (23.0)	−2.39	0.017	0.782
Ferritin (ng/mL)	20.0–275.0	1139.3 (1772.9)	1490.1 (1815.7)	−0.52	0.603	0.117
Transferrin (mg/dL)	200.0–360.0	132.0 (31.7)	136.0 (68.0)	−0.82	0.410	0.269
Glucose (mg/dL)	75.0–115.0	154.0 (81.0)	184.5 (113.5)	−1.62	0.106	0.328
Total Cholesterol (mg/dL)	140.0–200.0	138.5 (51.5)	159.0 (103.0)	−2.02	0.044	0.574
GOT or AST (U/L)	5.00–40.0	37.0 (32.5)	31.0 (32.0)	−1.76	0.078	0.351
GPT or ALT (U/L)	0.00–55.0	35.0 (40.0)	36.5 (46.5)	−1.21	0.228	0.248
LDH (U/L)	0.00–248.0	490.5 (183.0)	429.0 (138.0)	−3.05	0.002	0.590
CRP (mg/L)	0.00–5.00	153.7 (210.7)	35.4 (56.4)	−4.66	0.001	0.991
PCT (ng/dL)	0.02–0.50	0.22 (0.44)	0.11 (0.46)	−1.59	0.112	0.360
Hemoglobin (g/dL)	11.0–17.0	13.3 (2.80)	12.6 (3.93)	−4.06	0.001	0.789
Leukocytes (*10^3^/μL)	3.50–10.5	9.67 (6.94)	9.45 (7.44)	−1.25	0.212	0.240
Neutrophils (%)	42.0–77.0	88.5 (8.15)	88.0 (6.82)	−0.34	0.737	0.067
Lymphocytes (%)	20.0–44.0	6.40 (5.68)	5.75 (4.13)	−0.28	0.777	0.056
Platelets (*10^3^/µL)	120.0–450.0	212.0 (135.5)	266.0 (131.5)	−2.52	0.012	0.482
D-dimer (ng/dL)	0.00–500.0	1080.0 (1647.5)	1520.0 (3050.0)	−1.14	0.254	0.231
Fibrinogen (mg/dL)	200.0–350.0	750.5 (356.5)	556.0 (336.7)	−3.52	0.001	0.683
Ca (mg/dL)	8.80–10.6	8.40 (0.48)	8.10 (0.98)	−0.81	0.421	0.190
P (mg/dL)	2.30–4.50	3.55 (1.93)	3.15 (1.43)	−0.02	0.984	0.005
Mg (mg/dL)	1.60–2.60	2.23 (0.37)	2.20 (0.50)	−0.80	0.421	0.253
25–OH–D (ng/mL)	20.0–100.0	13.6 (9.02)	12.2 (6.01)	−2.53	0.011	0.600
25–OH–D_3_ (ng/mL)	-	8.45 (6.38)	7.92 (5.85)	−1.35	0.176	0.520
25–OH–D_2_ (ng/mL)	-	5.85 (2.95)	4.66 (2.02)	−2.74	0.006	0.278

*N* = 37. Data expressed as median (interquartile range, IQR). Abbreviations: SD = standard deviation; ES = effect size; bpm = beats per minute; brpm = breaths per minute; PEEP = positive end-expiratory pressure; PaO_2_/FiO_2_ = partial oxygen arterial pressure/fraction of inspired oxygen; GOT or AST = glutamic oxaloacetic transaminase or aspartate transaminase; GPT or ALT = glutamic pyruvic transaminase or alanine transaminase; LDH = lactate dehydrogenase; CRP = C-reactive protein; PCT = procalcitonin; Ca = calcium, P = phosphorous, Mg = magnesium, *10^3^ = multiplied by 1000. The sixth column reports statistical significance after the Wilcoxon signed-rank test; evolution is shown after 3 days. ES effect size calculations were also made to determine the effect of ICU stay (ES: small ≤ 0.01, moderate = 0.06, and large ≤ 0.14) [28]. Statistical significance = *p* < 0.05.

**Table 3:** Matrix for correlation coefficients (rho) showing the simple linear relationship between clinical and biochemical parameters with 25-OH-D, 25-OH-D_2_, and 25-OH-D_3_ levels.

	Baseline			Follow-Up		
	25-OH-D (ng/mL)	25-OH-D_3_ (ng/mL)	25-OH-D_2_ (ng/mL)	25-OH-D (ng/mL)	25-OH-D_3_ (ng/mL)	25-OH-D_2_ (ng/mL)
Age (years)	−0.129	−0.132	−0.035	−0.089	−0.132	0.065
BMI (kg/m^2^)	0.111	0.165	−0.026	0.091	0.261	−0.231
APACHE-II	−0.103	−0.066	−0.040	-	-	-
SOFA	−0.154	−0.053	−0.167	−0.133	0.008	−0.290
Respiratory rate (brpm)	−0.176	0.053	−0.456 ^a^	−0.277	0.047	−0.374
Albumin (g/dL)	0.403 ^a^	0.285	0.390 ^a^	0.015	−0.097	0.234
PCT (ng/dL)	−0.270	−0.294	−0.026	−0.587 ^b^	−0.458 ^a^	−0.331
Hemoglobin (g/dL)	0.387 ^a^	0.307	0.261	0.301	0.223	0.224
Lymphocytes (%)	−0.046	−0.059	0.084	0.364 ^a^	0.371 ^a^	0.034
D-dimer (ng/dL)	−0.521 ^b^	−0.644 ^b^	0.148	−0.264	−0.405 ^a^	0.302
Fibrinogen (mg/dL)	0.350 ^a^	0.370 ^a^	0.128	0.116	0.335	−0.521 ^b^
Ca (mg/dL)	0.285	0.180	0.306	−0.333	−0.527 ^b^	0.426 ^a^

Matrix correlations are presented as correlation coefficients (rho). Abbreviations: BMI = body mass index; APACHE-II = Acute Physiology and Chronic Health Evaluation II; SOFA = Sequential Organ Failure Assessment; FiO_2_ = fraction of inspired oxygen; PCT = procalcitonin; Ca = calcium. Statistical significance ^a^ = *p* < 0.05; ^b^ = *p* < 0.01.

## Data Availability

The datasets generated and analyzed during the current study are not publicly available because the database is very extensive and includes data from other studies complementary to this but are available from the corresponding author on reasonable request.
